# Mechanisms governing avian phylosymbiosis: Genetic dissimilarity based on neutral and MHC regions exhibits little relationship with gut microbiome distributions of Galápagos mockingbirds

**DOI:** 10.1002/ece3.6934

**Published:** 2020-10-27

**Authors:** Ramona Fleischer, Alice Risely, Paquita E. A. Hoeck, Lukas F. Keller, Simone Sommer

**Affiliations:** ^1^ Institute of Evolutionary Ecology and Conservation Genomics University of Ulm Ulm Germany; ^2^ Zoological Museum University of Zurich Zurich Switzerland; ^3^ Department of Evolutionary Biology and Environmental Studies University of Zurich Zurich Switzerland

**Keywords:** biogeography, host–microbe interactions, major histocompatibility complex, microsatellites, *Mimus* sp., wildlife genetics

## Abstract

The gut microbiome of animals, which serves important functions but can also contain potential pathogens, is to varying degrees under host genetic control. This can generate signals of phylosymbiosis, whereby gut microbiome composition matches host phylogenetic structure. However, the genetic mechanisms that generate phylosymbiosis and the scale at which they act remain unclear. Two non‐mutually exclusive hypotheses are that phylosymbiosis is driven by immunogenetic regions such as the major histocompatibility complex (MHC) controlling microbial composition, or by spatial structuring of neutral host genetic diversity via founder effects, genetic drift, or isolation by distance. Alternatively, associations between microbes and host phylogeny may be generated by their spatial autocorrelation across landscapes, rather than the direct effects of host genetics. In this study, we collected MHC, microsatellite, and gut microbiome data from separate individuals belonging to the Galápagos mockingbird species complex, which consists of four allopatrically distributed species. We applied multiple regression with distance matrices and Bayesian inference to test for correlations between average genetic and microbiome similarity across nine islands for which all three levels of data were available. Clustering of individuals by species was strongest when measured with microsatellite markers and weakest for gut microbiome distributions, with intermediate clustering of MHC allele frequencies. We found that while correlations between island‐averaged gut microbiome composition and both microsatellite and MHC dissimilarity existed across species, these relationships were greatly weakened when accounting for geographic distance. Overall, our study finds little support for large‐scale control of gut microbiome composition by neutral or adaptive genetic regions across closely related bird phylogenies, although this does not preclude the possibility that host genetics shapes gut microbiome at the individual level.

## Graphical abstract

This study uses the Galápagos mockingbird species complex to test the relationship between gut microbiome composition and host genetic structure at the population level based on both MHC and microsatellite genotypes. We found no relationship between microbiome composition and genetic distance based on either MHC or microsatellite markers. This suggests that, at this geographic scale, host population genetics exerts little influence on the gut microbiome.

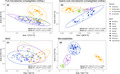

## 1 INTRODUCTION

The gut microbiome of animals is a source of both functionality and pathogens, and is to various degrees under host genetic control (Davenport, 2016; Kubinak et al., 2015; Kurilshikov et al., 2017). However, the mechanisms by which host genetic variation shapes gut microbiota composition and the scale at which they act remain unclear (Kohl, 2020; Kurilshikov et al., 2017; Lim & Bordenstein, 2020). Within host species, pairwise similarity in taxonomic microbiome composition between individuals has been found to correlate with host genetic similarity (e.g., Griffiths et al., 2018, 2019; Kohl et al., 2017; Smith et al., 2015; Suzuki et al., 2019; Webster et al., 2018), yet generally the effect of genetics on the microbiome within species is thought to be modest (Kurilshikov et al., 2017) and not always detectable (e.g., Rothschild et al., 2018; Yuan et al., 2015). The influence of host genetics becomes stronger at cross‐species phylogenetic scales, with large‐scale studies demonstrating considerable host phylogenetic effects on the gut microbiome, although such affects are weaker in birds than mammals (Amato et al., 2018; Song et al., 2020; Trevelline et al., 2020; Youngblut et al., 2019).

A number of non‐exclusive mechanisms could underpin the influence of host genetics on gut microbiome composition. Adaptive genetic diversity (i.e., genes or loci that affect host fitness and are therefore under selection; Holderegger et al., 2006) may be disproportionately important in controlling the gut microbiota. For example, genetically encoded components of the immune and endocrine system regulate the gut microbiome (Donaldson et al., 2018; Noguera et al., 2018), and variation in these regions could have disproportionally large effects on microbiota composition compared with neutral regions. One region of particular interest is the major histocompatibility complex (MHC), a highly diverse suite of genes that are crucial to the adaptive immune system across vertebrates (Piertney & Oliver, 2006; Sommer, 2005). Variation in the MHC has been repeatedly linked to host microbiome composition (Bolnick et al., 2014; Kubinak et al., 2015; Leclaire et al., 2019; Pearce et al., 2017; Silverman et al., 2017), and this regulation by the MHC may be adaptive by protecting against autoimmunity (Silverman et al., 2017) and reducing host susceptibility to infection (Kubinak et al., 2015). Spatial variation in parasite and pathogen communities across the host species range may therefore select for population‐specific compositions in MHC genotypes that have the potential to generate divergent gut microbiome communities across the host population.

Alternatively, or in addition to MHC effects, differences in neutral host genetic diversity (genes or loci that have no effect on host fitness and are therefore not under selection; Holderegger et al., 2006) may contribute to the phylogenetic and spatial structuring of gut microbiomes observed both within and across species. While studies that link neutral gene markers to the microbiome are still uncommon in wild populations, there is evidence that variation in neutral regions of the host genome within species is associated with microbial composition of gut (Smith et al., 2015), skin (Griffiths et al., 2018), and coral surfaces, even in the presence of high gene flow (Griffiths et al., 2019). As host species diverge, the accumulation of such neutral effects via founder effects, genetic drift, or isolation by distance, would together generate a distinct phylogenetic signal on the host‐associated microbiome.

While both neutral and adaptive genetic regions have been implicated in being important for shaping gut microbial communities, the extent to which these mechanisms act together has not been explicitly tested. Moreover, disentangling host genetic effects from environmental effects is statistically challenging since host‐associated microbiomes, host genetics, and landscape characteristics (e.g., climate, soil properties, vegetation) are often spatially autocorrelated. Both host‐associated microbiomes and host genetics are subject to isolation by distance due to limited dispersal of both hosts and microbes (Martiny et al., 2006; Moeller et al., 2017), potentially generating spurious correlations where no direct link exists (Grieneisen et al., 2019). Mantel tests and their derivatives (e.g., multiple regression on distance matrices; MRMs) are a common method in microbiome analysis to test for correlations between genetic and ecological variation across spatially structured populations. However, Mantel tests suffer from a number of statistical drawbacks and can generate false positives (Legendre et al., 2015). Recently, a Bayesian inference method called *BEDASSLE* that was developed to account for spatial autocorrelation when testing the contribution of ecological variables to spatially structured genetic data (Bradburd et al., 2013), was applied to microbiomes (Grieneisen et al., 2019), showing that this method can be used to understand drivers of gut microbiome variation across spatially distributed host populations.

In this study, we combine island‐level data on gut microbial species diversity, MHC allele composition, and microsatellite diversity (as a measure of neutral genetic variation and phylogenetic distance) across four Galápagos mockingbird species to test the relative contribution of neutral and adaptive genetic markers in shaping the gut microbiome composition. The Galápagos mockingbirds comprise four generalist species distributed allopatrically across almost all islands of the Galápagos Archipelago, diverging into four genetically divergent clades approximately 500,000 (95% credible interval: 145,957–1,388,173) years ago after a single colonization event (Nietlisbach et al., 2013). Previous studies have found that genetic drift has acted to reduce genetic diversity of mockingbirds compared with other Galápagos bird species (Hoeck et al., 2010) and that the MHC is under selection across islands and species (Vlček & Štefka, 2020), making the mockingbird complex a particularly good study system to investigate the role of host genetics in shaping gut microbiomes.

We combine gut microbiome data, MHC, and microsatellite data collected largely from different individuals; therefore, we use island averages to test whether island populations that are genetically similar also tend to be more similar in their gut microbiomes. If host genetics tends to have large effects on the gut microbiome, we would expect correlations between genetic similarity and microbiome similarity between islands. Effects of genetic variation on the gut microbiome at the individual level require paired samples collected from the same individual and therefore cannot be detected by this study. Nevertheless, community‐wide genetic effects, whereby allele frequencies across islands drive differences in overall gut microbiome composition, are detectable within this framework.

Here, we apply both MRM tests and *BEDASSLE* to test for the effects of neutral and MHC dissimilarity while controlling for geographic distance. Since phylogenetic signals in gut microbiomes have been shown to be weak yet detectable in avian clades (Kropáčková et al., 2017; Song et al., 2020; Trevelline et al., 2020), including Galápagos finches (Michel et al., 2018), we expect that adaptive immune genetic regions may have larger effects on microbiome composition than neutral markers in this study system.

## 2 MATERIAL AND METHODS

### 2.1 Study sites, species distribution, and sample sizes

**FIGURE 1:**
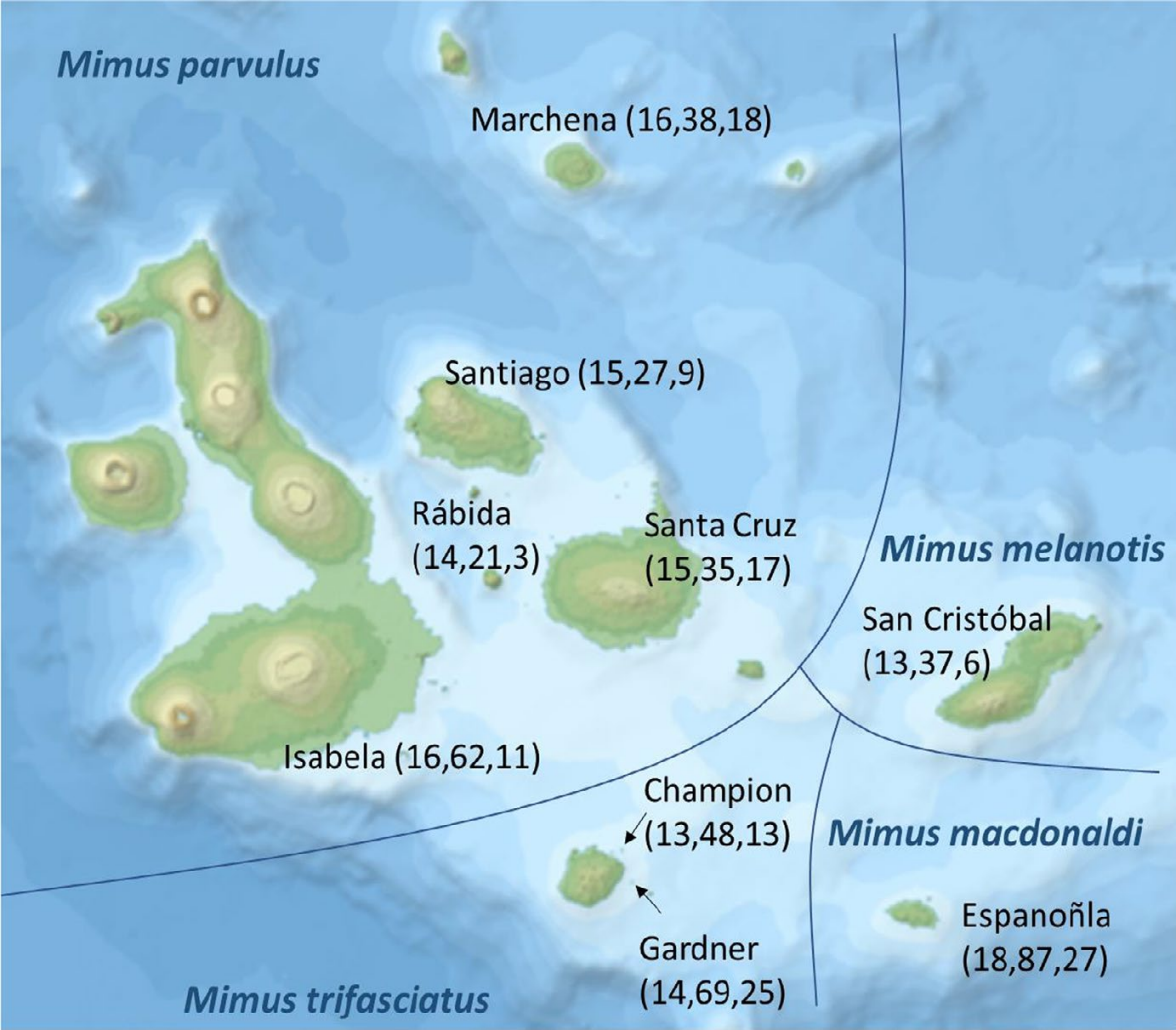
Map of the Galápagos Archipelago with species distributions, sample locations, and sample sizes. The numbers indicate the number of individuals sampled per island for MHC (first number), microsatellites (second number), and microbiome (third number)

Four endemic mockingbird species (*Mimus parvulus*, *M. trifasciatus*, *M. macdonaldi*, and *M. melanotis*) that allopatrically inhabit islands of the Galápagos Archipelago were sampled between 2004 and 2008 as part of one overarching study. Blood samples were collected from 2004 to 2008 and used for MHC and microsatellite genotyping; fecal samples were collected largely from different individuals (overlap of 31 individuals between blood and fecal samples) between 2006 and 2008. Sample metadata is listed in Table S1. We compared nine island populations (Isabela, Marchena, Santiago, Santa Cruz, Rábida, Champion, Gardner, San Cristóbal, and Española, Figure 1), for which data on microsatellite genetic diversity and differentiation (Hoeck et al., 2010) and MHC divergence (Vlček et al., 2016) were already available, and generated the microbiome data in the present study. Blood and fecal samples were not necessarily obtained from the same individual birds but from the same islands and species; therefore, we focus on island averages of genetic parameters and gut microbiome composition in our analysis.

### 2.2 Assessment of neutral and adaptive genetic variation, and gut microbial species diversity per island

Variation in microsatellite genotypes among 135 individuals, based on 16 microsatellite loci, was estimated using the R package *adegenet* (Jombart, 2008) and visualized in a principal component analysis (PCA) including the first two axes that together explained 36% of variation. To calculate mean pairwise distances between islands, we generated an individual‐based genetic distance matrix based on Nei's distance using the nei.dist() function in package *Poppr* (Kamvar et al., 2014) and calculated mean pairwise distance between islands using meandist() function in the package *vegan* (Oksanen et al., 2007).

We downloaded MHCII exon 2 B sequence data for 177 birds from Vlček et al. (2016). To estimate genetic distance between 119 MHC alleles, we generated a tree based on the genetic similarity of the whole MHC allele sequence using the packages *ape* (Paradis & Schliep, 2019) and *phangorn* (Schliep, 2011). This tree was rooted using an MHC allele sequence of a related species (*M. polyglottos*), which was afterward removed from the final tree. We then calculated pairwise dissimilarity of allele composition between all individuals per island, also taking allele genetic similarity into account, using the distance() function in *phyloseq* (McMurdie & Holmes, 2013), and calculated mean distance between islands using *vegan* package's meandist() function (Oksanen et al., 2007). While we also generated MHC supertypes to assess functional variation, these resulted in only seven supertypes, too few to calculate distances between islands (data not shown).

The microbiome data were derived from fecal samples of 129 birds. DNA was extracted from frozen fecal pellets and amplified using the paired primers 515F/ 806R for amplification of the V4 region of the bacterial 16S ribosomal RNA (16S rRNA) gene. Paired‐end sequencing of the amplicons was performed with Illumina MiSeq technology over 2 × 250 cycles. The paired‐end sequencing results were processed in QIIME 2 (Bolyen et al., 2019) using the DADA2 pipeline (Callahan et al., 2016). Before analysis, we excluded amplicon sequence variants (ASVs) that were not assigned to the kingdom bacteria (261), not assigned at phylum level (253), and assigned to chloroplasts (136) or mitochondria (3,017). All singletons (25,500) were excluded because they are likely to represent artefacts (Kopylova et al., 2016), but they represented only 3.4% of total reads. Alpha diversity was measured with three indices that emphasize different features of the individual microbial communities: the observed ASV richness which represents the count of ASVs, the Simpson index which takes into account the abundance of ASVs, and the Faith's phylogenetic diversity (PD) index which takes into account the phylogeny of ASVs. We calculated beta diversity based on unweighted UniFrac (which is based on presence/absence and controls for taxa phylogeny; Lozupone et al., 2011) and weighted UniFrac (which additionally considers abundance) and used multidimensional Scaling (MDS) based on the distance matrix for visualizing beta diversity. We then extracted mean distance between island population centroids using the meandist() function in *vegan* (Oksanen et al., 2007). For beta diversity, samples were rarefied to a minimum sequence depth of 10,000 to normalize for sequencing depth (Weiss et al., 2017).

For statistical analyses, we generated two microbiome datasets, one representing the overall microbiome, excluding rare taxa with a prevalence of under 5% (leaving 1,228 ASVs), and one representing the island common core (each island microbiome dataset was filtered separately so that only taxa that had over 50% prevalence remained). We also applied 30% and 70% prevalence thresholds for the island core with no difference to the results. The island common core was applied to accentuate the differences in common gut microbes between islands, and because any effects of host genetics may act on local microbial communities. We excluded low prevalence taxa in the full microbiome dataset because they are difficult to model statistically and to limit computational time for the *BEDASSLE* analysis. However, sensitivity analyses with expanded number of ASVs did not change the results (Methods S1).

### 2.3 Statistical modeling

We conducted analyses at both the individual level (to assess variation within each genetic layer) and island level (to test for associations between microbiome composition and host genetics). We estimated the effects of species and island on dissimilarity in gut microbiota ASV distributions (full microbiome and island core), genetic dissimilarity based on MHC allele frequencies, and genetic dissimilarity based on microsatellite markers, respectively, by using PERMANOVA tests on individual distance matrices described above, presenting standardized effect sizes (SES).

To assess the effects of MHC and microsatellite dissimilarity on microbiome composition at the average island level, we used two methods: First, we applied multiple regressions on distance matrices (MRM) that predict mean pairwise distance of microbiome composition between islands, using the package *ecodist* (Goslee & Urban, 2007). MRMs are a derivative of the Mantel test but allow multiple variables within the model. We included mean geographic distance between islands, as well a variable we term “comparison type,” which codes whether the islands being compared are inhabited by the same species or different species, to control for these factors. Because the number of island populations being compared was small (*n* = 9 islands, generating 36 pairwise comparisons), there is a risk of overfitting the models, and model selection on MRMs using AIC generates spurious results (Franckowiak et al., 2017). Therefore, we present all possible models in Table S2a (predicting full microbiome) and Table S2b (predicting island core microbiome) and assess support for each variable across models. Statistics presented for PERMANOVAs and MRMs all predict microbiome dissimilarity based on unweighted UniFrac. Beta diversity plots based on weighted UniFrac are visualized in the supplementary material.

In addition to MRMs, which are prone to statistical problems and have low statistical power (Legendre et al., 2015), we applied a Bayesian method called *BEDASSLE* (Bayesian estimation of differentiation in alleles by spatial structure and local ecology), which was developed to quantify the individual effects of geographic distance and ecological variables on population genetic structure (Bradburd et al., 2013). *BEDASSLE* has recently been applied to microbiomes (Grieneisen et al., 2019) and quantifies the geographic shift in microbiome distributions (in km) represented by a one‐unit increase in the predictor variable (in this case, MHC and microsatellite genetic distances). If the effect size of microsatellites was 500 km, the interpretation would be that a one‐unit shift in microsatellite genetic distance has the impact of approximately 500 km of extra pairwise geographic distance on gut microbiome distributions. The results should therefore be interpreted in the context of the Galápagos Islands, which span 270 km and have a mean distance between islands of about 80 km. For context, human populations separated by the Himalayas display genetic differences equivalent to 11–16,000 km lateral distance (Bradburd et al., 2013). We applied a beta‐binomial model within the *BEDASSLE* R package to predict the occurrence of 1,228 ASVs and included distance matrices representing normalized mean pairwise dissimilarity in MHC and microsatellite markers between island populations as covariables, following the methodology of Grieneisen et al. (2019). We repeated this analysis for the island core microbiome (267 ASVs), which we ran for 20 million generations. Further information on *BEDASSLE* and its implementation is described in Supplementary Methods. All R code and data can be downloaded at https://github.com/Riselya/Mockingbird‐microbiome‐paper.

## 3 RESULTS

**FIGURE 2:**
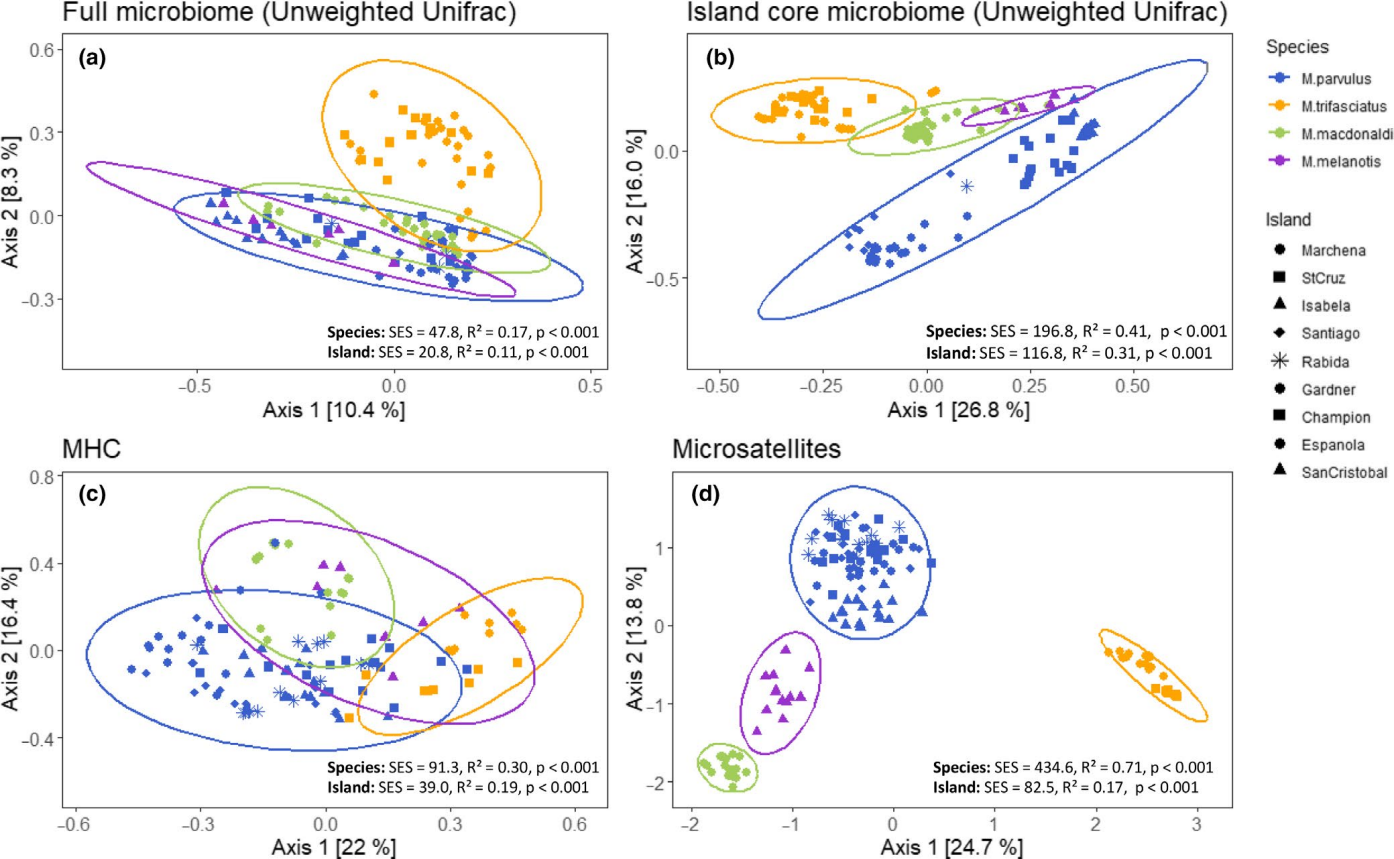
Ecological dissimilarity between species and islands for (a) the overall microbiome; (b) the island core microbiome; (c) MHC genotypes; and (d) microsatellite genotypes. Points represent sampled individuals and are colored by species and shaped by island. SES, standardized effect size

We first assessed variation in gut microbiome, MHC, and microsatellite at the individual level to examine the effects of species and island on the three levels of genetic data separately. Gut microbiome diversity demonstrated large interindividual variation (Figure S1). Alpha diversity was largely similar across species, though *M. melanotis* tended to have slightly lower diversity across all measures (Figure S2). Beta diversity clustered weakly by species and island when including all taxa (Figure 2a; see Figure S3 for weighted UniFrac). As expected (because the island core microbiome was generated per island), beta diversity clustered strongly by both species and island for the island core microbiome (Figure 2b). MHC allele composition exhibited somewhat stronger clustering by species and by island than the full microbiome (Figure 2c), while, in accordance with earlier results (Hoeck et al., 2010), microsatellite profiles showed the strongest clustering by species and more moderate clustering by island (Figure 2d).

**FIGURE 3:**
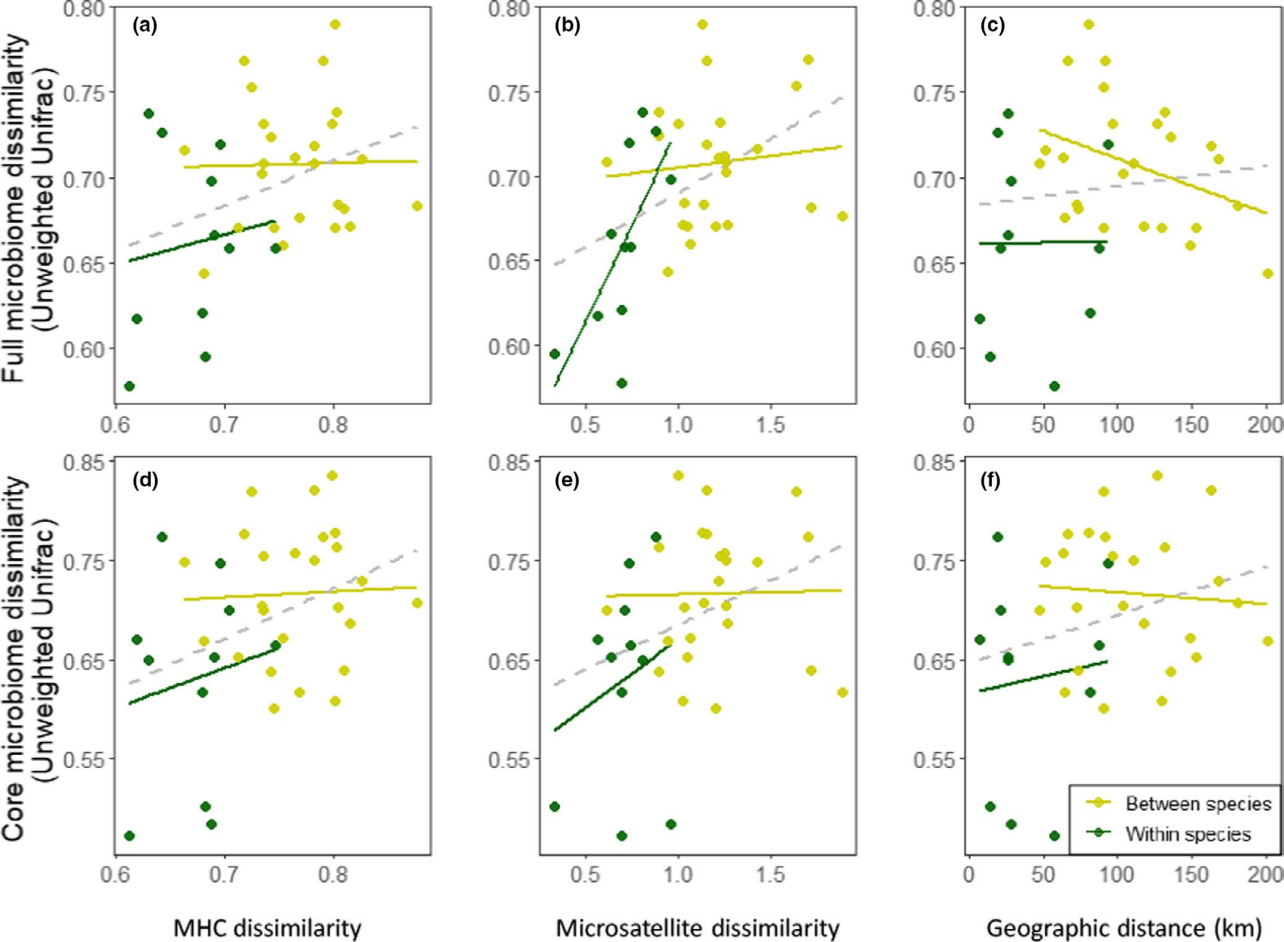
(a–c) Pairwise dissimilarity between overall microbiome beta diversity and (a) MHC dissimilarity; (b) microsatellite dissimilarity; and (c) geographic distance. Bottom panel (d–f) shows pairwise distances between the island core microbiome and (d) MHC dissimilarity; (e) microsatellite dissimilarity; and (f) geographic distance. Each point represents the mean pairwise distance between two islands and is colored by whether the comparison is within species (dark green) or between species (light green). The gray dashed line shows the overall trend (statistics found in Table S2 ).

We then examined associations between island centroids for the three layers of data. We estimated the effects of MHC and microsatellite dissimilarity on both the full microbiome (Figure 3a–c) and island core microbiome (Figure 3d,e) composition using both MRM tests, controlling for geographic distance and comparison type (within/between species) and *BEDASSLE*. Genetic distances based on both MHC and microsatellite dissimilarity significantly correlated with gut microbiome dissimilarity in univariate MRM tests for both overall and island core microbiomes (Figure 3a,b,d,e; Table S2a,b, respectively). Across multivariate MRM models (models 5–15 Table S2a,b), however, there was little support for the effect of MHC distance on overall microbiome or island core composition (Figure 3a; Table S2a,b), and some support for the effect of microsatellite distance on overall microbiome composition (Table S2a) but little for island core microbiome composition (Table S2b). We noted a significant correlation between MHC and microsatellite data when applying Mantel tests, and both of these variables were also correlated with geographic distance (MHC—microsatellite: Mantel *R* = 0.49, *p* = .004; MHC—geographic distance: Mantel *R* = 0.58, *p* = .004; microsatellite—geographic distance: Mantel *R* = 0.28, *p* = .002; Figure S4).

To effectively control for landscape effects, we additionally applied *BEDASSLE* to estimate the relative contribution of MHC and microsatellite distances on gut microbiome distributions (1,228 ASVs with over 5% prevalence). We found that a one‐unit increase in microsatellite genetic distance (i.e., one‐unit increase in microsatellite Euclidean distance) shifted microbiome distributions by the equivalent of 45.4 km (Table 1a). When this effect size was normalized to fit the distribution of our microsatellite data (0.69–1.22; see Figure 2d), this was reduced to 24.1 km (Table 1a, right side panel). Given that the Galápagos islands span 270 km, and the mean distance between islands is 80 km, 24.1 km effectively denotes an effect size of zero. The effect of MHC distance (i.e., one‐unit increase in MHC Euclidean distance) was predicted to have zero effect on gut microbiome distributions. We repeated this analysis on island core microbiomes (267 ASVs) and found similarly small normalized effects of microsatellite distance (4.2 km) and MHC distance (5.1 km).

**TABLE 1:** BEDASSLE effect sizes for the effect of microsatellite and MHC distances on (a) the full microbiome and (b) the island core microbiome

Predictor variables	Mean geographical distance equivalent (km) per 1 unit predictor variable (95% CI)	Effect size for each predictor (in km) normalized to reflect the difference between the 25th and 75th percentiles in our microsatellite and MHC data
(a) Full microbiome		
Microsatellite	45.4 (34.7–56.4)	24.1
MHC	6.1 (4.5–8.0)	0.6
(b) Island core microbiome		
Microsatellite	8.3 (0–20.1)	4.2
MHC	42.9 (19.0–76.0)	5.1

Note

The first panel shows effect sizes per one‐unit increase in the predictor variable, while the second panel normalizes this to reflect the middle 50% of the data.

## 4 DISCUSSION

This study aimed to quantify the effects of genetic dissimilarity based on host neutral and MHC allele frequencies on the gut microbiome ASV distributions of the Galápagos mockingbird species complex at the island population level. Our results support other studies that find relatively weak correlations between neutral genomic diversity and microbiome composition (e.g., Griffiths et al., 2019; Kohl et al., 2017; Smith et al., 2015; Suzuki et al., 2019; Webster et al., 2018). Analyses with *BEDASSLE* suggest that this correlation is a consequence of spatial autocorrelation between host genetic and microbiome clustering, rather than a direct consequence of host genetics acting upon gut microbial communities. Similar results were observed regarding the influence of MHC dissimilarity on microbiome distributions, contradicting our prediction that adaptive genetic regions may exert stronger influence on the gut microbiome than neutral markers in avian systems. Our results suggest that neither genetic differentiation based on neutral nor adaptive markers appear to exert detectable control over gut microbiome distributions in Galápagos mockingbirds at the population level. The weak clustering of gut microbiomes across species and island observed in this system is therefore likely to be the result of biogeography alone. Our results highlight that spatial autocorrelation between ecological and genetic variables due to limitations in both host and microbe dispersal (e.g., Moeller et al., 2017) may lead to an overestimate of the link between host genetics and microbiome composition.

Our results add to the increasing number of studies showing that phylogenetic signals in bird gut microbiomes are relatively weak (Michel et al., 2018; Song et al., 2020; Trevelline et al., 2020). They also suggest that in a species complex with a limited species range, and where pathogen diversification might be relatively low, regions of the genome that reflect adaptation to local pathogen‐driven selection pressures, such as the MHC, are rather unimportant in explaining the diversification of the gut microbiome. These results may not apply to mammalian lineages or to other bird phylogenies covering larger biogeographic ranges. Mammals show much stronger signals of phylosymbiosis than birds, and demonstrate phylogenetic signals in their gut microbiomes even when accounting for geographic distance (Amato et al., 2018; Kartzinel et al., 2019). Nevertheless, the development of methods that account for spatial autocorrelation between host genetics and microbiomes due to isolation by distance acting on both host and microbe communities may improve estimates of their direct relationship across host phylogenies, including mammals.

Our study applies microbiome and genetic data from different individuals, therefore is not able to detect genetic effects on the microbiome at the individual level. Paired samples, where gut microbiome, microsatellite, and MHC data all come from the same individual, would allow for more sophisticated statistical methods (e.g., redundancy analyses or joint species distribution modelling) with increased power to detect weak effects and associations between species alleles and microbial taxa, thereby helping to elucidate underlying mechanisms driving phylosymbiosis. For example, mockingbird species may share genetic elements that strongly affect the microbiome yet would not be detected here. Our results therefore do not preclude that host genetics influences gut microbe distributions in this system, yet suggest that if such control exists it is likely to be targeted towards specific microbial genera, governed by genetic regions not assessed in this study, or only detectable at larger phylogenetic scales. MHC polymorphism has been associated with the abundance of a limited number of microbial families (Bolnick et al., 2014; Kubinak et al., 2015), suggesting its influence may be obscured by environmental effects, or ecological differences in life history (such as differences in diet and food availability across or even within islands). Nevertheless, our study mirrors the results from other Galápagos bird species that show only weak clustering of species or islands microbiome(Michel et al., 2018), which indicates that any effects of host genetics on overall microbiome composition are likely to be weak at this phylogenetic scale. Our study confirms this lack of association at the island population level in a species complex with well‐documented population genetics.

This study does not test for the effects of specific MHC alleles on gut microbiome distributions, and we note that specific MHC alleles are likely to have targeted effects on the gut microbiome. The detection of such effects remains challenging where genetic effects are weak and numbers of populations examined are necessarily limited by species range and structure. In this study, we examined nine genetically isolated populations across four closely related species, with numbers of populations in other studies ranging from five to 15 (Griffiths et al., 2018, 2019; Kohl et al., 2017; Smith et al., 2015; Suzuki et al., 2019; Webster et al., 2018). Limited statistical power may therefore to some degree explain a lack of robust associations between genetics and microbiomes, especially for nonmammalian clades for which phylosymbiosis appears relatively weak. We also found that MHC and neutral dissimilarity were correlated in this study, which may also limit statistical power to distinguish between the two. Such correlations are common in small populations where genetic drift can act on both neutral and adaptive alleles (Radwan et al., 2010; Zeisset & Beebee, 2014). Therefore, while our results suggest no broad effects of genetics on gut microbiome distributions, weak or targeted effects of host genetics on the microbiome that scale up with biogeographic distance and host phylogeny may not have been detected within this study design.

## 5 CONCLUSIONS

Our study brings together three layers of genetic data to test the relative contribution of neutral (microsatellite) and adaptive (MHC) genetic regions in shaping gut microbiome diversity in Galápagos mockingbirds. Whileour study is necessarily limited by the number of mockingbird populations, our study is the first to simultaneously test the independent contributions of neutral and MHC dissimilarity on gut microbiome distributions across a well‐studied avian species complex. Our results support previous studies finding weak effects of phylogeny on bird gut microbiomes, yet go further to suggest that weak correlations between host genetics and microbiomes in allopatric populations may be a consequence of spatial autocorrelation between these factors. Future research that investigates the targeted effects of host genetics on specific microbial lineages within individuals may elucidate the relationship between avian genetics and the gut microbiome at a finer‐scale resolution.

## CONFLICT OF INTEREST

No authors have any conflicting interests in regard to this paper.

## AUTHOR CONTRIBUTION

**Ramona Fleischer:** Conceptualization (supporting); Formal analysis (equal); Investigation (lead); Methodology (lead); Writing‐original draft (equal); Writing‐review & editing (equal). **Alice Risely:** Conceptualization (equal); Formal analysis (equal); Supervision (equal); Validation (equal); Writing‐original draft (lead); Writing‐review & editing (equal). **Paquita E. A. Hoeck:** Data curation (lead); Investigation (supporting); Methodology (supporting); Writing‐review & editing (equal). **Lukas Fridolin Keller:** Data curation (lead); Investigation (supporting); Methodology (supporting); Writing‐review & editing (equal). **Simone Sommer:** Conceptualization (equal); Resources (lead); Supervision (lead); Writing‐review & editing (equal).

## ETHICAL APPROVAL

Samples were collected with the permission of Galápagos National Park Service (permit number PC‐29‐05).

## Supporting information

Fig S1‐S4

Table S1

Table S2

Methods S1

## Data Availability

Data and code for this manuscript are publicly available to download at https://github.com/Riselya/Mockingbird‐Microbiome‐Phylosymbiosis‐Project. Microbiome sequences are available under project PRJNA658437 on NCBI. MHC and microsatellite data are downloadable from the original studies.
