# Supplementary material for: Mechanisms governing avian phylosymbiosis: Genetic dissimilarity based on neutral and MHC regions exhibits little relationship with gut microbiome distributions of Galápagos mockingbirds
Source: Ecol Evol. 2020 Oct 27;10(23):13345–54. doi: 10.1002/ece3.6934 (PMC7713960; doi:10.1002/ece3.6934)
Supplement: Supplementary file 1 — Fig S1‐S4 [file ECE3-10-13345-s001.docx]

# **SUPPLEMENTARY FIGURES**

**Supplementary Figure 1**


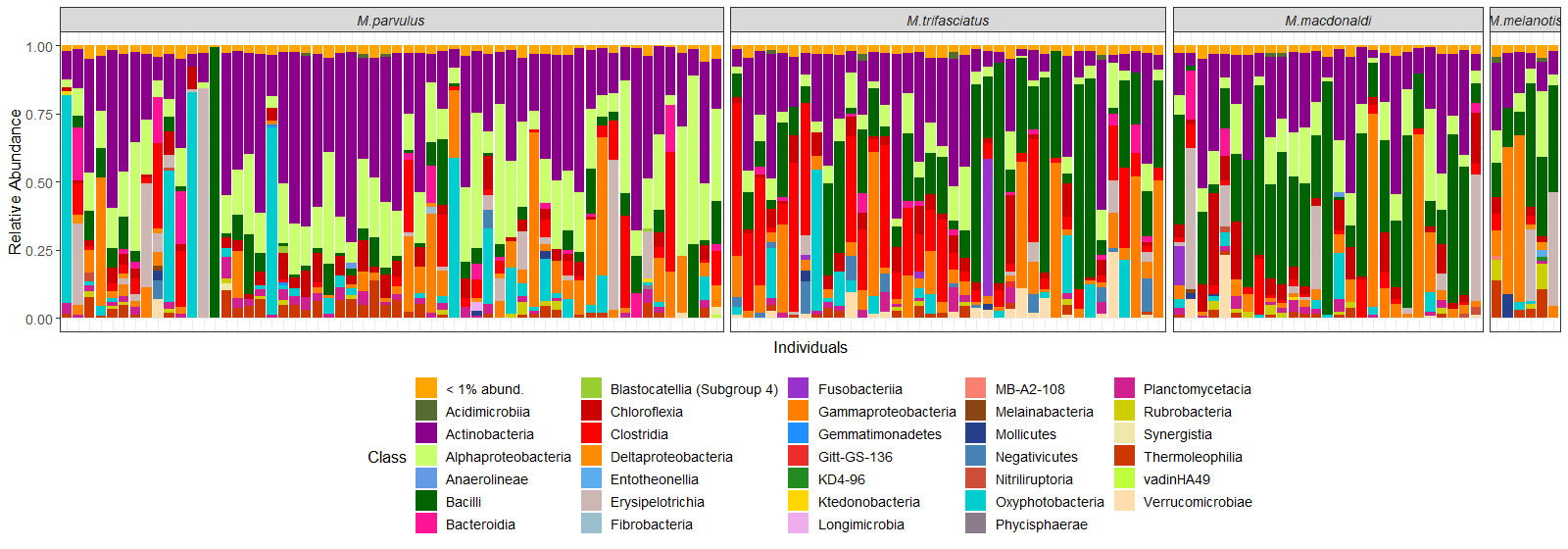


Figure S1) Individual composition of gut bacteria at the class level grouped by mockingbird species. Each colour refers to a bacterial class. Bacterial classes with abundance below 1% are condensed into one single group.

**Supplementary Figure 2**


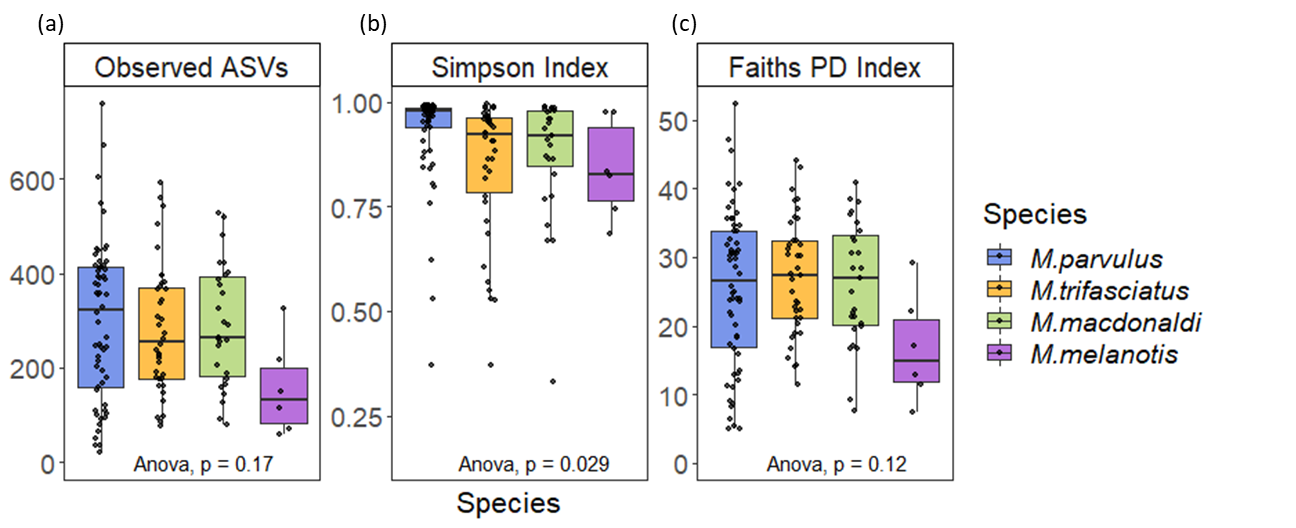


Figure S2) Gut microbiome alpha-diversity scores of (a) observed ASV richness, (b) Simpson Index and (c) Faiths PD Index for each mockingbird species, including all ASVs. Each dot represents one individual bird, p-values of ANOVA tests are reported at the bottom of each panel.

**Supplementary Figure 3**


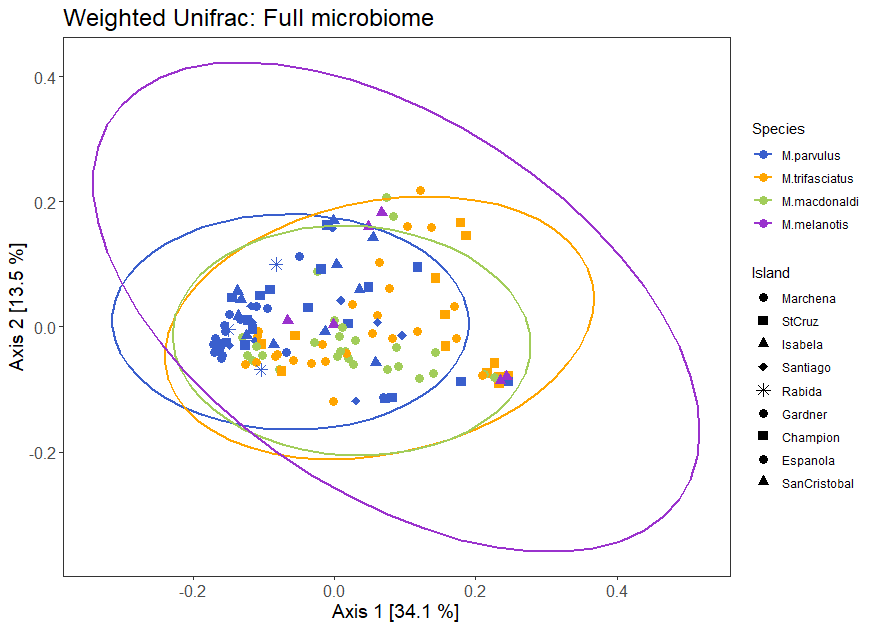


Figure S3) Gut microbiome beta-diversity including all bacterial taxa, measured by Weighted UniFrac distances, including all ASVs (Species: SES = 20.6, R² = 0.15, p = 0.001; Island: SES = 7.1, R² = 0.09, p = 0.001). Each dot represents one individual; dots are coloured by species and shaped by island.

**Supplementary Figure 4**


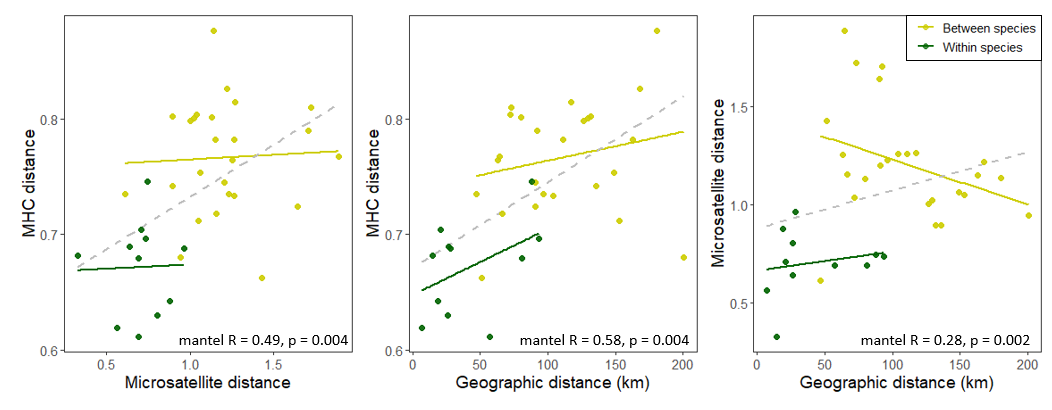


Figure S4) Pairwise correlations between the tested variables: a) MHC and microsatellite genotype, b) MHC and geographic distance, c) and microsatellite genotype and geographic distance. Each dot represents one pair of islands. Colours represent whether the comparison is within (dark green) or between (light green) species. Trend lines are included for both groups in the respective colours, and also for all island comparisons as dashed grey line. The mantel R statistic and p-values are reported for the overall correlation (i.e. grey dashed line).
