# Supplementary material for: Mechanisms governing avian phylosymbiosis: Genetic dissimilarity based on neutral and MHC regions exhibits little relationship with gut microbiome distributions of Galápagos mockingbirds
Source: Ecol Evol. 2020 Oct 27;10(23):13345–54. doi: 10.1002/ece3.6934 (PMC7713960; doi:10.1002/ece3.6934)
Supplement: Supplementary file 4 — Methods S1 [file ECE3-10-13345-s004.docx]

**Supplementary Methods**

**BEDASSLE ANALYSIS**

BEDASSLE was originally developed to model the independent effect of various ecological distances to population genetic structure, whilst accounting for population spatial structure. This method was recently applied to gut microbiome data, and was robust to sensitivity analyses and model assumptions (Grieneisen *et al.* 2019). Here we repeat the methods applied in Grieneisen et al. (2019) and apply a beta-binomial model to 1228 ASVs (presence/absence) that had over 5% prevalence across all individuals. We ran the final model for 15 million generations with a 60% burn-in, and then ran 1,000 posterior predictive sample replicates to evaluate model fit (Fig. M1 below). We ran sensitivity analyses by clustering ASVs by their phylogenetic relatedness (applying tip_glom() in the package *Phyloseq;* n = 496 ASVs), with no significance difference to model results. In addition, we ran it on ASV’s with over 2% prevalence (n = 3100 ASVs), again with similar results, suggesting the model was robust to changes in ASV processing methods. Lastly, we changed the delta shift parameter by several orders of magnitude to test whether this affected model inference, with little impact to model results.

We also applied the same model to the island core microbiome (n = 167 ASVs) . representing ASVs that were in at least 50% of samples from each island. We ran this model for 20 million generations to allow parameter stabilization (Fig. M2). Code is available at <https://github.com/Riselya/Mockingbird-Microbiome-Phylosymbiosis-Project>.


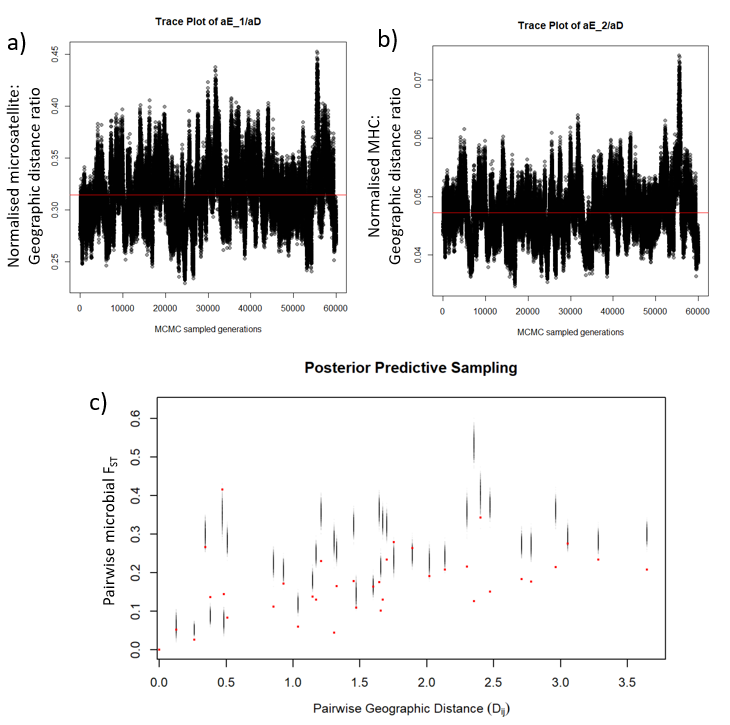


**Figure M1)** BEDASSLE output graphs for the model predicting overall gut microbiome distributions (n = 1228 ASVs). The BEDASSLE model was run with geographic distance and microsatellite and MHC FST normalized by their standard deviations. A) Ratio of normalised microsatellite distance to geographic distance after a 60% burn-in; b) Ratio of normalised MHC distance to geographic distance after a 60% burn-in; c) Predicted distributions of microbial Fst (black lines) verses observed data (red dots). This suggests the model tends to overestimates microbial distances in some cases. Given we report an effect size of zero, we consider that improved model fit is highly unlikely to change overall interpretation.


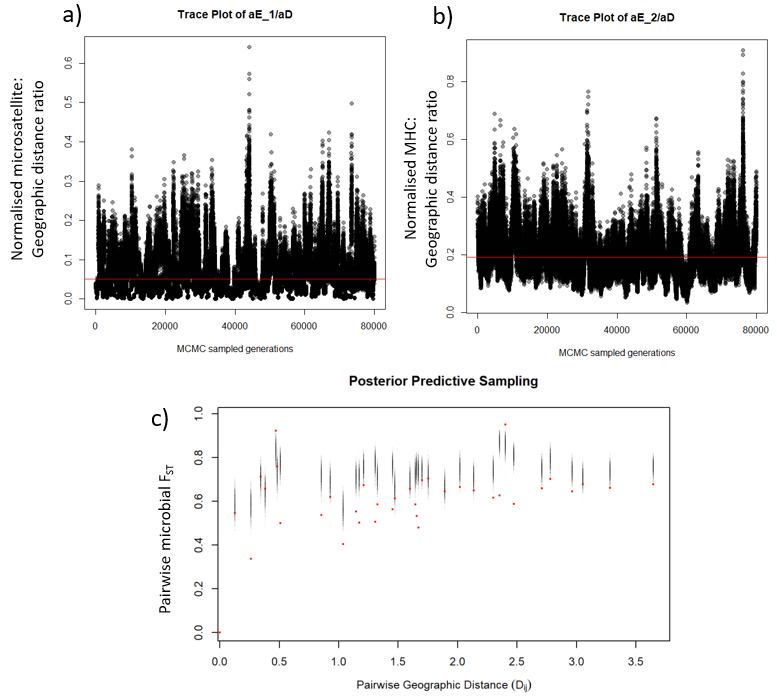


**Figure M2)** BEDASSLE output graphs for the model predicting island core microbiome distributions. The BEDASSLE model was run with geographic distance and microsatellite and MHC FST normalized by their standard deviations. A) Ratio of normalised microsatellite distance to geographic distance after a 60% burn-in; b) Ratio of normalised MHC distance to geographic distance after a 60% burn-in; c) Predicted distributions of microbial F_ST_ (black lines) verses observed data (red dots). This suggests relatively little variation in predicted microbial F_ST_ for core microbiome distributions.

References

Grieneisen, L.E., Charpentier, M.J., Alberts, S.C., Blekhman, R., Bradburd, G., Tung, J. & Archie, E.A. (2019) Genes, geology and germs: gut microbiota across a primate hybrid zone are explained by site soil properties, not host species. *Proceedings of the Royal Society B,* **286,** 20190431.
